# National partnerships address critical needs in infection prevention and control

**DOI:** 10.1017/ash.2024.447

**Published:** 2024-12-02

**Authors:** Erica Kaufman West, Michelle Doll, Margaret A. Fitzpatrick, James Lewis, Priya Nori, Catherine Passaretti, Dalilah Restrepo, Michael P. Stevens, Rama Thyagarajan, Catriona Hong

**Affiliations:** 1 Department of Infectious Diseases, Franciscan Alliance, Munster, IN, USA; 2 Department of Medicine, Virginia Commonwealth University School of Medicine, Richmond, VA, USA; 3 Department of Medicine, Division of Infectious Diseases, University of Colorado School of Medicine, Aurora, CO, USA; 4 Division of Allergy and Infectious Diseases, Department of Medicine, University of Washington, Seattle, WA, USA; 5 Snohomish County Health Department, Everett, WA, USA; 6 Department of Medicine, Division of Infectious Diseases, Montefiore Health System, Albert Einstein College of Medicine, Bronx, NY, USA; 7 Department of Infection Prevention, Division of Quality, Advocate Health, Charlotte, NC, USA; 8 UCI-Los Alamitos Hospital, Orange County, CA, USA; 9 Division of Infectious Diseases, West Virginia University School of Medicine, Morgantown, WV, USA; 10 Department of Internal Medicine, Dell Medical School at University of Texas at Austin, Austin, TX, USA; 11 University of Connecticut School of Medicine, Farmington, CT, USA

## Abstract

The COVID-19 pandemic highlighted gaps in infection control knowledge and practice across health settings nationwide. The Centers for Disease Control and Prevention, with funding through the American Rescue Plan, developed Project Firstline. Project Firstline is a national collaborative aiming to reach all aspects of the health care frontline. The American Medical Association recruited eight physicians and one medical student to join their director of infectious diseases to develop educational programs targeting knowledge gaps. They have identified 5 critical areas requiring national attention.

## Introduction

The COVID-19 pandemic highlighted gaps in infection control knowledge and practice across health settings nationwide. The Centers for Disease Control and Prevention (CDC) recognized that all frontline health care professionals need targeted infectious diseases education to keep themselves, their families, coworkers, and patients safe. With funding through the American Rescue Plan, CDC partnered with organizations with a regional or national scope, who could reach all aspects of the frontline—environmental services workers, nurses, facility engineers, physicians, and more. CDC also recognized that with infectious diseases, there are disparate outcomes associated with historically marginalized and under-resourced groups (such as people from racial and ethnic minority groups, those in rural areas, and others) that need to be addressed.

The American Medical Association’s mission “to promote the art and science of medicine and the betterment of public health” rendered it a natural partner. The AMA, founded in 1847, is the largest association of physicians and medical students in the United States and is therefore in a unique position to reach physicians and physicians-in-training across multiple specialties in various professional stages. Ensuring physicians and trainees receive essential education in infection prevention and control (IPC) is a priority.

To begin its work, the AMA conducted listening sessions, diagnostic interviews and surveys with physicians to hear what they needed from Project Firstline efforts. Then, the AMA recruited eight physicians and one medical student to join their director of infectious diseases to critically appraise IPC knowledge gaps across the country and act as subject matter experts. These ten individuals have years of IPC experience and represent diverse clinical settings from nine of the ten U.S. Department of Health and Human Services regions.

This work group recognizes that the U.S. health system has many challenges: lack of universal access to health care leaves patients struggling to pay medical bills, a physician workforce that has improved in diversity but not enough to represent the population at large, workforce shortages and burnout, limited health care access in rural areas, and hospital closures due to financial distress are just a few. IPC is impacted by each of these challenges, and yet is often overlooked and underfunded. One specific goal of this work group was to highlight these challenges to the larger public health community and to individual physicians and patients by delineating high priority targets for improved IPC knowledge and practice across the spectrum of frontline health care professionals.

### Specific priorities

The work group has itemized the following gaps as priorities for its work and for other groups invested in IPC. These gaps were identified repeatedly during monthly meetings at which iterative discussions were held among work group members and consensus was reached that these topics necessitated a large-scale response.

1. Seasonal respiratory illnesses
2. Staffing
3. Equity
4. Medical education
5. Environmental impact

## Seasonal respiratory illnesses

The COVID-19 pandemic has altered the way we view health risks associated with respiratory viral infections. In health care, universal masking was implemented as an evidence-based source control measure to reduce the risk of nosocomial SARS-CoV-2 transmission among patients and health care professionals (HCP).^1,2^ The end of the World Health Organization and the U.S. federal government COVID-19 Public Health Emergency declarations^1,2^ has prompted discussions around the future of masking in health care settings^3,4^ as COVID-19 is increasingly viewed as an “established and ongoing health issue.”^5^

Regardless of individual perceptions regarding the ongoing health implications of COVID-19, our pre-pandemic approach towards preventing hospital-acquired respiratory viral infections was likely inadequate, and we underappreciated the harm caused by hospital acquired respiratory viral infections preventable by masking.^6^ Lessons learned from the COVID-19 pandemic have led to a greater appreciation of asymptomatic, presymptomatic, and pauci-symptomatic transmission of SARS-CoV-2 and other respiratory viruses^4,7^ from patients to HCPs and vice versa. Additionally, pre-pandemic policies assumed patients’ respiratory infections were identified and isolated appropriately, but studies have shown that asymptomatic and presymptomatic cases likely account for most SARS-CoV-2 transmission events^8^; the same may be true for other respiratory viruses.^9,10^ Thus, reverting to pre-pandemic interventions will not prevent transmission in the health care setting.

While masking is an evidence-based intervention to prevent the transmission of respiratory viruses in direct person-to person encounters, a misinterpretation of the oft-cited Cochrane Review,^11^ which was a population level study suggesting masking is not effective at stopping the spread of COVID-19 at the community level, was inappropriately and incorrectly extrapolated to health care facilities.

It is also important to note that one cost of masking is the barrier to clear communication in clinical encounters. Physicians should demand wider access to items such as OSHA-compliant clear masks that enable those with hearing impairments to participate in their care without sacrificing safety. In addition, masking recommendations impacting large sections of the health care system must be balanced against HCP and patient PPE fatigue and the resulting difficulties in attaining compliance.

### Next steps in masking

Varying approaches to integrate masking as part of routine health care policies could be considered beyond the pandemic. Masking could be implemented 1) across health care spaces year-round; 2) in targeted high-risk settings, such as transplant, oncology, and geriatric units; 3) in specified months during the local respiratory viral season; or 4) when community burden of respiratory viruses approaches a critical threshold.^4^ For example, in Washington State, regional health care organizations issued a living joint consensus statement^12^ in April 2023 to extend universal masking in patient care spaces of health care facilities. The most recent iteration of this statement utilized Emergency Department discharge diagnosis syndromic surveillance data as a surrogate for community respiratory virus transmission burden for influenza, RSV, and COVID-19^13^ to advocate for a threshold approach. When thresholds for transmission are surpassed, it triggers universal masking in health care settings for affiliated organizations. This multisystem approach allows facilities to address patient safety through a collaborative and supportive approach that accounts for regional variations in community respiratory viral burden, while allowing flexibility to tailor policies to unique spaces. Reporting on the impact of regional interventions on nosocomial transmission will be important. In addition, facilities can use their own employee infection data, which is faster to obtain and arguably most relevant to their staff, to make decisions on masking policies.

## Staffing

**Figure 1.**
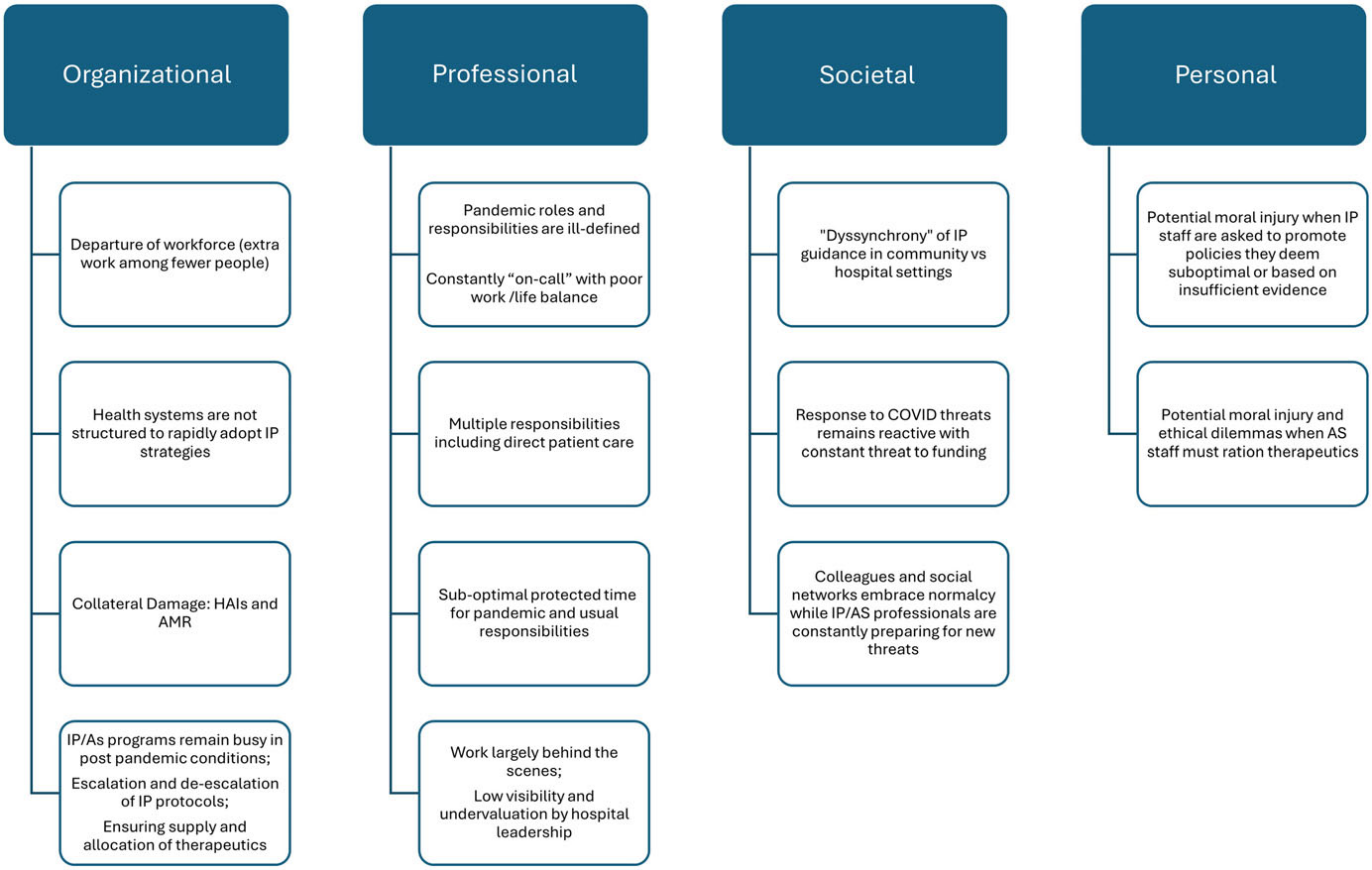
Complex factors contributing to Infection Prevention burnout (reproduced with permission from Antimicrobial Stewardship & Healthcare Epidemiology).

The COVID-19 pandemic led to significant burnout in the infection prevention workforce.^14^ However, even prior to this, IPC programs were understaffed. A 2019 national survey revealed 25% of programs had at least one open position and that it took 3 to 6 months to recruit trained infection preventionists (IPs). Additionally, 40% of IPs are expected to retire over the next decade.^15^ Burnout and the decision to leave IPC occurs for a myriad of reasons, as outlined by Nori et al. (Fig. 1).^14^

Critically, IPC staffing support models of 1 IP to every 75–100^16^ acute care beds are outdated and do not consider an evolving scope of practice.^17^ New staffing models must account for expanded responsibilities, like oversight of infection prevention of hemodialysis centers, same day surgery centers, outpatient clinics, etc. Regarding long-term care facilities (LTCFs), Centers for Medicare and Medicaid Services (CMS) requires at least part-time on-site IPC staffing that “must meet the needs of the facility.” However, the reality is that LTCFs are generally understaffed, may have four-fold fewer IPs than hospitals and suffer from high staff turnover, which has been significantly worsened recently.^18–20^ Staffing models that reflect these realities are needed to ensure adequate IPC infrastructure in LTCFs.

There is also a paucity of trained health care epidemiologists (HCE) in the United States. HCEs should be trained in Infectious Diseases (ID), a specialty facing critical shortages. It is estimated that 79.5% of U.S. counties do not have an ID physician.^21^ Forty-four percent of ID fellowship positions were unfilled in the 2022 Match,^22^ and there is currently no accredited certification in health care epidemiology.

### Next steps in staffing

Innovative models for recruiting and training both IPs and HCEs are desperately needed. In the absence of a dramatic expansion of ID physicians, creative approaches to expanding the reach of existing HCE should be considered, such as expansion of telehealth consultation or the elevation of HCE to system-level positions. In a recent survey of ID fellowship program directors, 96% supported a formal certification program in HCE with the majority recommending that it not require additional years of training.^23^ Additionally, expansion of HCE to include non-ID trained physicians may be needed, ideally supported by formal training and certification.

## Equity

The importance of health care equity extends to IPC, antimicrobial stewardship and emerging pathogen response. Part of health care equity is ensuring equitable access to the same level of care; however, antimicrobial stewardship (AS), IPC, and infectious diseases resources can vary widely between types of settings. Compared to urban acute care facilities, rural settings often lack AS pharmacist support and access to an ID physician. In addition, rural IPs often have fewer years of experience, limited access to high-quality/comprehensive infection prevention education, are less often to be certified in IPC, and are more likely to have additional non-IPC responsibilities.^24^ Similarly, despite the shift to more complex patients and increasing numbers of invasive procedures in non-acute settings,^25^ experienced infection prevention and stewardship staff remain limited^26^ and staffing benchmarks are unclear in ambulatory, skilled nursing and home-care settings. Antimicrobial use and prescribing practices also have been shown to vary by geographic region, with the southeast issuing the most antimicrobial prescriptions.^27^ While the reasons for disparate outcomes are multiple, differences in IPC and AS staffing, training and education likely contribute, and data on disparities in health care associated infections (HAIs) and antimicrobial resistance outcomes are just emerging.

IPC and AS outcomes vary by type of facility. Past studies have suggested that smaller hospital size was associated with higher central line associated bloodstream infection (CLABSI) rates.^28^ During the COVID-19 pandemic, smaller community hospitals had more marked increases in HAIs compared to academic hospitals.^29^ Safety net hospitals have higher CLABSI, catheter associated urinary tract infection, and post-colectomy surgical site infection rates compared to non-safety net hospitals.^30^

Studies also show variability in HAIs by patient demographic characteristics, with higher rates of health care associated bloodstream infections in Black and Hispanic patients compared to white patients.^31–36^ Racial differences exist for *Staphylococcus aureus* infections,^37^ *Clostridioides difficile* infections^38^ and frequency of antibiotic prescriptions. Social vulnerability, those determinants that adversely affect a community, are receiving more attention as risk factors for adverse health outcomes, including HAIs. A higher social vulnerability index (SVI)^39,40^ and limited English language proficiency have similarly been associated with worse infection outcomes.^33,41,42^ Nevertheless, data suggests many HCPs are unaware of impact of health disparities on HAIs and other health outcomes.^43^

### Next steps in equity

Improved education and knowledge of health care disparities are essential to providing safe care for all. Project Firstline is working to address knowledge gaps through free educational materials. In addition, institutions should look at their patients’ social vulnerabilities when collecting HAI data to see if certain populations require more education or interventions. In addition, NSHN could start requiring SVI and other health equity metrics within their data reporting modules to encourage more research into this area.

## Medical education

Education must emphasize the evidence supporting IPC practices and promote long-term compliance with repeated short bursts of engagement to hardwire behavioral change. Knowledge of and compliance with evidence-based IPC practices are required by health care professionals at all levels. However, given the AMA’s priority to ensure physicians and trainees received essential education in IPC, the work group focused on education for these groups.

Medical school is usually the first formative exposure to IPC for physicians. Online training modules for the prevention of infections and environmental hazards are specifically required by the Liaison Committee on Medical Education (LCME),^44^ but medical schools are otherwise not required to cover IPC in their curricula. Basic concepts like hand hygiene are reinforced through simulated clinical encounters^45^; however, a lack of consensus exists on the optimal content and delivery of IPC education in medical school. Multiple studies have shown that medical students have knowledge gaps regarding foundational IPC practices such as hand hygiene, donning/doffing PPE, HAIs, and transmission-based risk assessment.^46–49^

Opportunities for IPC education in residency and fellowship are more clinically oriented and guided by the Accreditation Council for Graduate Medical Education (ACGME) Infectious Diseases Milestones for IPC and Antimicrobial Stewardship and Epidemiology.^50^ These milestones help determine whether a trainee achieves competency in IPC knowledge and practices, and suggest tools to assess competency.^51–53^ However, they do not indicate specific curricular content, discuss which assessment models and tools are most effective, or consider how education and training should be adapted for different settings. Several studies have identified pervasive knowledge gaps among residents and fellows regarding IPC practices, such as preventing spread of antimicrobial resistance.^54^ In one study, only half of ID fellows in the greater New York area felt comfortable managing everyday IPC scenarios,^49^ and a large survey study of U.S. Pediatric ID fellows showed that less than half participated in a dedicated IPC rotation.^55^ A recent survey of ID Fellowship program directors revealed that only one-third have a dedicated IPC/HCE tract, and the average formal didactic hours on IPC topics over the course of the fellowship is less than 5 hours. Directors cited lack of funding, lack of time and lack of a formal curriculum as reasons their programs have not expanded education on this topic.^23^

Finally, upon completion of medical training, practicing clinicians may receive minimal continuing education in IPC practices. Many hospitals require completion of online IPC training modules for credentialing, but education is sporadic, may not be engaging, and may address only superficial IPC knowledge. While professional societies and CDC offer courses, the utilization and effectiveness of these resources is largely unknown.

CDC, through Project Firstline and other sources, has educational tools available in various formats. While sources like those developed by the Association for Professionals in Infection Control and Epidemiology (APIC) provide highly specific educational tools for IP specialists, many tools are relevant to all health care professionals (Table 1).

**Table 1.** Examples of IPC education and training resources available to health care professionals

Source	Type of educational resource	Target audience	Link
CDC Project Firstline Resources			
Micro Learns	Short, adaptable, guided IPC discussions for on-the-job training available in multiple formats (PDF, PPT)	Front-line health care professionals (HCP)	Training Toolkits from Project Firstline | Infection Control | CDC
Training toolkits	Live, web-based training sessions with groups of health care workers to recognize infection risks throughout their workday	Front-line HCPs Facilitator guides available for leaders without extensive IPC background	
Other IPC Education Materials	Interactive activities and infographics, videos and other social media content, print materials and job aids	Front-line HCPs	Access Educational Materials from Project Firstline | Infection Control | CDC
Project Firstline Facebook	Updates on new training or resources with links	Front-line HCPs	https://www.facebook.com/CDCProjectFirstline General public

Other CDC resources			
CDC/CMS Nursing Home Infection Preventionist Training	Free online training course with modules and exam	Nursing home and long-term care facility staff	Infection Prevention Training | LTCF | CDC
CDC States Targeting Reduction in Infections via Engagement (STRIVE) Infection Control Training	Written content and materials to lead educational courses for new employees, annual IPC trainings, and periodic training	Frontline HCPs IPC staff, hospital administration, EVS, patient and family advisors	STRIVE Infection Control Training | CDC

Selected resources from other organizations			
Agency of Healthcare Research and Quality (AHRQ) Healthcare Associated Infections Program	Comprehensive subject-based toolkits including print and online content and resources	Clinicians, administrators, informaticists, information technology staff	AHRQ’s Healthcare-Associated Infections Program | Agency for Health care Research and Quality
Statewide Program for Infection Control and Epidemiology	Education and consultation services for North Carolina and beyond	Frontline HCPs IPC Staff Clinicians	SPICE
World Health Organization Infection Prevention and Control (IPC) Technical and Clinical Hub	Guidelines and publications on various IPC topics, free online training courses via Open WHO IPC Channel	Frontline HCPs globally (materials offered in many languages)	Infection prevention and control (who.int)
Association of Professionals in Infection Control (APIC)	Written content and infographics; online courses, webinars, CE, and micro learns	Clinicians, IPC staff General public	Online Learning—APIC infectionpreventionandyou.org
Society for Healthcare Epidemiology of America (SHEA)	Webinars, virtual networking events, workshops, online training courses	Frontline HCPs ID fellows	LearningCE @ SHEA
Infection Prevention and Control Canada (IPAC)	Online training courses, e-learning tools, webinars, scholarships, and grants	Frontline HCPs IPC staff	Home | IPAC Canada
American Medical Association (AMA)	Tele-mentoring sessions*, webinars, podcasts*, CME module	Physicians, medical trainees, frontline HCPs	AMA Project Firstline

* The AMA work group contributed to the development of physician-targeted educational resources as part of the AMA’s collaboration with CDC on Project Firstline.

### Next steps in education

Effective education should offer multiple methods like short videos, infographics, case-based learning, and interviews with subject matter experts along with traditional articles and didactics. Additionally, we must ensure the tools reach target audiences. Furthermore, to better engage learners, IPC educational content should include evidence behind best practices, areas that need more research, and frame all IPC activities as patient safety interventions.

Project Firstline’s Community College collaboration is an example of how early IPC educational exposure addresses unique training needs of a diverse, intergenerational health care workforce. For medical students, an expanded, longitudinal collaboration with the LCME to develop consistent and standardized IPC curricula could improve IPC education at this stage. New, innovative learning tools should ensure that key IPC content is covered in and out of classrooms to create permanent behavior change.

## Environmental impact

The health care industry produces 4%–5% of the world’s greenhouse gasses,^56^ with the U.S. health care system contributing 8.5% of the nation’s greenhouse gas emissions.^57^ Climate change can exacerbate pathologies and expand the geography of infectious diseases. The work group felt that the dichotomy between understanding that IPC is important to protect patients and professionals from infection while acknowledging the impact that IPC policies have on health care’s contribution to climate change required scrutiny.

During the pandemic, the consumption of single use PPE skyrocketed in health care and among the public. Increases in waste emerged from test kits, chemical waste, vaccine by-products (syringes, needles, and safety boxes),^58^ heightened PPE policies, and disinfection.

Prior to the pandemic, the worldwide utilization of facemasks was about 89 million per month, which increased to 129 billion per month during the pandemic,^59^ with proper disposal still an area of ongoing research. One study^60^ estimated that in the U.S. alone, a new N95 respirator per patient encounter would require 7.41 billion respirators, cost $6.38 billion, and generate 84.0 million kilograms of waste over the course of only 6 months.^60^

However, health care professionals are taking an active role in environmentally conscious policies. For instance, the Green Surgery Report^61^ released on November 14, 2023, details next steps to create more environmentally sustainable clinical practices. Such practices include limiting use of PPE to when it is clearly indicated and prioritizing handwashing over disposable gloves during routine medical care to reduce carbon emissions and potential microorganism transmission. The report also calls for more research on reusable devices, with specific consideration for IPC.

In-home, hospital, clinical and public space disinfecting practices have also increased significantly since the COVID-19 pandemic. Disinfectants contain chemicals such as quaternary ammonium compounds, hydrogen peroxide, bleach, and alcohols, which have significant negative consequences on the environment^62^ and water supplies.^63^ Waste treatment centers are not specifically designed to handle these compounds.^64^ The Environmental Protection Agency,^65^ the World Health Organization (2020),^66^ and CDC (2021)^67^ have guidance for cleaning and disinfectants but none address environmental concerns.

### Next steps in environmental impact

It is important that the environmental impact of IPC guidance be acknowledged. Experts should appraise existing guidelines for PPE, contact precautions and institutional disinfectant practices. Agencies can support research to identify more environmentally friendly alternative PPE options and medication, vaccine and equipment packaging and dispersal. Collaboration with the Environmental Protection Agency and Occupational Health and Safety Administration might inform ways to limit disinfectant pollution to water systems and the environment. Finally, institutional efforts should be supported to improve sustainability through designated committees charged with reducing the carbon footprint of direct patient care and non-patient care activities.

## Conclusion

The COVID-19 pandemic has produced numerous opportunities to improve the outlook of IPC in the U.S. Working proactively to identify infectious diseases threats and responding broadly ensure enhanced protection for our patients and health care workforce and provide health care parity across all facility settings. Acknowledging disparities and working to ensure every patient has access to equitable care and striving for equal access to educational opportunities for HCPs is crucial, as is ensuring that IPC education is a priority at all stages of training. Finally, it is our responsibility to recognize the environmental toll of our health care practices and take necessary steps to correct it.

The AMA’s Project Firstline Faculty Work Group has assembled a group of experts who are dedicated to ensuring the future of IPC. Through collaboration with other groups of thoughtful, committed individuals, real change can occur.
